# Beyond One-Size-Fits-All Active Surveillance for Low-Risk Prostate Cancer: Risk-Adapted Follow-Up, De-Escalation Pathways, and Focal Therapy as Tailored Strategy

**DOI:** 10.3390/diagnostics16091310

**Published:** 2026-04-27

**Authors:** Fabio Zattoni, Andrea Mari, Ugo Giovanni Falagario, Riccardo Giuseppe Bertolo, Simone Albisinni, Daniele Amparore, Lorenzo Bianchi, Riccardo Campi, Roberto Contieri, Elisa De Lorenzis, Paolo Dell’Oglio, Michele Marchioni, Veronica Mollica, Marco Moschini, Francesco Soria, Michele Talso, Filippo Turri, Savio Domenico Pandolfo

**Affiliations:** 1 Urology Clinic, Department of Surgery, Oncology, and Gastroenterology, University of Padua, 35128 Padua, Italy; 2 Department of Experimental and Clinical Medicine, University of Florence—Unit of Oncologic Minimally-Invasive Urology and Andrology, Careggi Hospital, 50134 Florence, Italy; 3 Department of Molecular Medicine and Surgery, Karolinska Institute, SE-171 76 Stockholm, Sweden; 4 Department of Urology, University of Foggia, 71122 Foggia, Italy; 5 Department of Urology, University of Verona, A.O.U.I., 37126 Verona, Italy; 6 Department of Urology, University of Tor Vergata, 00133 Rome, Italy; 7 Department of Oncology, University of Turin, Orbassano, 10043 Turin, Italy; 8 Division of Urology, Department of Surgery, Candiolo Cancer Institute, FPO-IRCCS, 10060 Candiolo (TO), Italy; 9 Division of Urology, IRCCS Azienda Ospedaliero-Universitaria di Bologna, 40138 Bologna, Italy; 10 Unit of Urology and Renal Transplantation, Department of Oncology, Careggi University Hospital, 50134 Florence, Italy; 11 Department of Experimental and Clinical Medicine, University of Florence, 50134 Florence, Italy; 12 Department of Urology, Istituto Nazionale Tumori “Fondazione Pascale”, 80131 Naples, Italy; 13 Department of Urology, IRCCS Foundation Cà Granda—Ospedale Maggiore Policlinico, 20122 Milan, Italy; 14 Department of Clinical Sciences and Community Health, University of Milan, 20122 Milan, Italy; 15 Department of Urology, ASST Grande Ospedale Metropolitano Niguarda, 20162 Milan, Italy; 16 UniCamillus—Saint Camillus International University of Health Sciences, 00131 Rome, Italy; 17 Department of Urology, Leonardo Foundation, 35037 Abano Terme, Italy; 18 Medical Oncology, IRCCS Azienda Ospedaliero-Universitaria di Bologna, 40138 Bologna, Italy; 19 Division of Experimental Oncology, Department of Urology, Urological Research Institute, Vita-Salute San Raffaele University, 20132 Milan, Italy; 20 Division of Urology, Department of Surgical Sciences—San Giovanni Battista Hospital, University of Studies of Torino, 10126 Turin, Italy; 21 ASST Fatebenefratelli—Sacco, Ospedale Fatebenefratelli, 20121 Milan, Italy; 22 Department of Urology, Fondazione Policlinico Universitario Agostino Gemelli IRCCS, Università Cattolica del Sacro Cuore, 00168 Rome, Italy; 23 Department of Life, Health and Environmental Sciences, University of L’Aquila, 67100 L’Aquila, Italy; 24 Department of Neurosciences, Reproductive Sciences and Odontostomatology, University of Naples “Federico II”, 80131 Naples, Italy

**Keywords:** low-risk prostate cancer, active surveillance, overtreatment, prostatectomy, focal therapy, quality of life

## Abstract

Low-risk prostate cancer (PCa) has historically been overtreated, exposing men to unnecessary morbidity. Emerging evidence supports conservative management of low-risk PCa without immediate radical intervention. Contemporary data show a marked decline in surgical overtreatment, with the proportion of radical prostatectomies yielding only Grade Group 1 cancers falling from 32.4% in 2010 to 7.8% in 2020 in the US SEER registry. Long-term studies confirm that deferring treatment is safe for low-risk disease, with PCa-specific survival exceeding 95% at 15–25 years for cohorts managed with surveillance. Major guidelines now endorse active surveillance (AS) as the preferred management for low-risk PCa. An alternative risk stratification system that expands the low-risk category was shown to reclassify 45–83% more men as low risk without increasing 15-year PCa mortality. Focal therapy has emerged as a potential middle-ground strategy, though evidence is still limited. The paradigm for managing low-risk PCa has shifted toward conservatism, with AS firmly established as the standard of care. Continued efforts to refine risk stratification and evaluate focal therapy are needed to further optimize individualized care, minimize harm, and maintain excellent cancer-specific outcomes for low-risk PCa. This comprehensive review aims to create a practical, risk-adapted framework for managing patients on AS. We will: (i) summarize inclusion criteria and outcomes, (i) compare AS follow-up schedules across major institutions and guidelines, (iii) provide evidence-based criteria to de-intensify surveillance in men with sustained stability and (iv) clarify the role of focal therapy as an intermediate treatment option within the AS continuum.

## 1. Introduction

Prostate cancer (PCa) remains a major cause of morbidity and can lead to premature death, particularly when clinically significant disease is diagnosed late. At the same time, the global burden is expected to increase substantially over the coming decades, largely driven by population ageing and growth, with forecasts suggesting a marked rise in new cases by 2040 [1].

Long-term randomized evidence indicates that organized PSA-based screening can reduce prostate cancer mortality, although at the cost of overdiagnosis and overtreatment when applied indiscriminately [2].

Low-risk PCa—typically defined as Gleason Grade Group 1 (GG1) (Gleason 3 + 3), PSA <10 ng/mL, and clinical stage T1–T2a—often follows an indolent course. Yet in past decades, many men with low-risk tumours received immediate radical prostatectomy or radiation. Such interventions provide minimal survival benefit in low-grade disease but can lead to substantial long-term side effects. Radical prostatectomy may cause urinary incontinence and erectile dysfunction [3,4,5], while radiation therapy (RT) can result in urinary and gastrointestinal complications, including radiation cystitis, chronic radiation proctitis, and sexual dysfunction. The recognition that many PCa are clinically insignificant drove a movement to reduce unnecessary treatment. Active surveillance (AS)—deferring definitive therapy while monitoring for signs of progression—has emerged as a strategy to preserve quality of life without compromising survival for low-risk patients [6].

Across contemporary international guidance, AS remains the preferred first-line strategy for men with GG1 PCa who have an appropriate life expectancy. The AUA/ASTRO guideline for clinically localized disease explicitly frames AS as the preferred management option in low-risk PCa, reinforcing its role as the standard of care rather than an “alternative” to treatment.

The EAU 2025 guideline similarly positions AS as a recommended option for ISUP/GG1 when other low-risk clinical features are present, aligning European practice with risk-adapted de-escalation.

Importantly, all major frameworks acknowledge that AS can be appropriate beyond GG1 in carefully selected favourable-intermediate presentations, provided adverse biological and pathological features are absent and disease burden is low.

Consistent with this, both EAU 2025 and NCCN v1.2025 support consideration of AS in selected favourable-intermediate cases—typically low-volume GG2/pattern 4 and otherwise favourable clinical parameters—while emphasizing rigorous selection and close follow-up to mitigate the higher progression risk relative to GG1.

With the present comprehensive review, we will summarize inclusion criteria and outcomes, biopsy follow-up schedules across major institutions and guidelines, discuss evidence-based criteria to de-intensify surveillance in men with sustained stability, and clarify the role of focal therapy as an intermediate treatment option within the AS continuum.

## 2. Materials and Methods

A comprehensive review was conducted by searching major databases, including PubMed/MEDLINE, Embase, Scopus, and the Cochrane Library, encompassing publications from January 2014 to September 2025. The aim of this approach was to provide a clinically oriented overview of the evolving management of low-risk prostate cancer.

### 2.1. Search Strategy and Study Selection

The search was structured around key terms pertinent to the objectives of the review. These included “low-risk prostate cancer”, “active surveillance”, “risk stratification”, “MRI”, “biomarkers”, “focal therapy”, and “overtreatment”. These keywords were selected to ensure a broad and comprehensive retrieval of relevant literature covering patient selection, diagnostic pathways, surveillance strategies, and emerging treatment approaches. Eligible items addressed localized low-risk prostate cancer (e.g., Grade Group 1, PSA ≤ 10 ng/mL, cT1–T2a or equivalent definitions) and included clinical trials, cohort studies, registry analyses, diagnostic studies, major guideline documents, and high-quality reviews. Non-English reports, animal studies, and small case series (<10 patients) were excluded.

Our review process aimed to include studies providing clinically meaningful insights into the management of localized low-risk prostate cancer, including randomized trials, large cohort studies, registry-based analyses, and major guideline documents.

To ensure a thorough and balanced selection, additional articles were identified through manual screening of reference lists from key studies, landmark trials, and relevant review articles.

Data extraction was performed independently by two authors, with a predefined strategy to resolve any discrepancies. In cases of disagreement, a third author was consulted to reach consensus. This collaborative approach ensured consistency and reliability in the interpretation of the evidence included in this review.

### 2.2. Data Synthesis

This review summarizes emerging trends and prospects in low-risk PCa. We searched MEDLINE/PubMed, Embase, Scopus, and the Cochrane Library from January 2014 to September 2025, supplemented by hand-searching references of key papers and guidelines. Eligible items addressed localized low-risk disease (e.g., GG1, PSA ≤ 10 ng/mL, cT1–T2a or near-equivalents) and included trials, cohort studies/registries, diagnostic studies, major guidelines, and high-quality reviews; non-English reports, animal studies, and case series (<10 patients) were excluded. The retrieved evidence was narratively synthesized to provide a clinically grounded overview of the current landscape of low-risk prostate cancer management, highlighting inclusion criteria for active surveillance, follow-up strategies, de-escalation pathways, and the emerging role of focal therapy. Evidence summaries were critically discussed and refined within a multidisciplinary expert panel (Uronauti group) to ensure a practice-oriented interpretation of the available data. Formal quantitative analysis was not performed. A detailed search strategy is reported in Table 1.

To facilitate clinical translation of the reviewed evidence, a risk-adapted management algorithm was developed and is presented. The flowchart integrates initial risk stratification, eligibility criteria for active surveillance and focal therapy, de-escalation pathways, and triggers for treatment escalation, providing a practical decision-making framework for clinicians managing low-risk Pca.

## 3. Discussion

### 3.1. Registry-Based Trends in Overtreatment and Active Surveillance

Recent large-scale cohort studies show significant declines in surgeries for indolent tumours, reflecting more judicious patient selection. Monda et al. (2025) [7] analysed pathology reports from two parallel registries, the U.S. SEER database and the Michigan Urological Surgery Improvement Collaborative (MUSIC), as a surrogate measure of overtreatment. They found a reduction in the fraction of prostatectomies that harboured only Gleason 6 cancer (pathologic GG 1) on final histology. Prostatectomies for low-grade tumours fell from 32.4% in 2010 to less than 8% by 2020. Similarly, statewide Michigan data revealed a reduction in pGG1 rates from 20.7% in 2012 to just 2.7% in 2024. However, these datasets include only men treated with prostatectomy and therefore cannot be interpreted as AS outcomes.

In a recent study including data on 26,999 men (2000–2016) on AS from the GAP3 registry [8], 10-year metastasis-free survival and overall survival were 99.4% and 84.1%, with lower rates of treatment conversion and longer treatment-free survival in more recent cohorts [8]. Such datasets are prone to substantial selection bias. Temporal changes in referral patterns, patient preferences, adherence to guidelines, and access to care may masquerade as improvement, without indicating that comparable populations were evaluated across time.

Definitions of “low-risk” disease and grading practice have evolved, producing grade migration across eras that can reclassify patients independently of true biology.

Moreover, the diffusion of mpMRI and targeted biopsy improved csPCa detection, reducing under-sampling and the risk of misclassification in the PRECISION study [9], which can make later cohorts appear safer and reduce low-grade diagnoses.

GAP3 additionally pools heterogeneous international cohorts with variable inclusion criteria, surveillance intensity, and triggers for intervention, limiting causal inference from time-period comparisons.

Incomplete capture of cause-specific outcomes (e.g., cause of death) and variable metastasis ascertainment across centres further constrain interpretation of “safety” endpoints in registry-based AS analyses.

In SEER/MUSIC, pGG1 prostatectomy is an informative system-level proxy for overtreatment, but it cannot adjudicate appropriateness in individual cases nor capture overtreatment driven by comorbidity or life expectancy.

Registry studies are also sensitive to missing data and coding changes over time; excluding incomplete cases can bias trends if missingness correlates with year or risk. Despite all these limitations, these prostatectomy-based and surveillance-based registries provide convergent evidence of improved risk stratification and increased acceptance of conservative management in low-risk disease.

### 3.2. Could Modernizing Risk Stratification Further Reduce Overtreatment?

The substantial decline in surgeries for low-grade PCa in favour of conservative management is strengthened by enhanced risk stratification methods, including advanced imaging and molecular biomarkers [10], which help differentiate between indolent and aggressive disease. While the “less is more” approach for low-risk PCa is gaining traction, current guidelines still rely on traditional classification parameters, suggesting a need for updated criteria that incorporate these modern diagnostic tools. Risk stratification models are evolving to better identify which patients truly need aggressive therapy and which do not.

A recent analysis of the ERSPC (European Randomized Study of Screening for Prostate Cancer) Rotterdam cohort examined whether modernizing risk stratification could further cut overtreatment [11]. The standard European Association of Urology (EAU) risk classification defines “low-risk” as any GG1 with PSA < 10 ng/mL and ≤cT2a; the study tested an alternative system that includes as low-risk any GG1 with PSA < 20 ng/mL and reclassifies certain favourable-intermediate risk patients as low-risk if they meet contemporary AS criteria (e.g., GG2 with PSA < 10 ng/mL and PSA density < 0.2 and ≤cT2a). Strikingly, this broadened definition increased the proportion of men labelled low-risk by 45% in the screening-detected cohort and 83% in the clinically detected cohort, compared to the traditional EAU criteria. Importantly, low-risk men according to the proposed criteria did not experience worse cancer-specific mortality compared to EAU low-risk men (subhazard ratio: 1.33 [95% CI: 0.66–2.68] in the screened group; 0.99 [95% CI: 0.29–3.38] in the unscreened group). In other words, many men currently classified as “intermediate-risk” by older criteria could safely be managed as low-risk, which would expand the pool of candidates for AS and further reduce overtreatment without compromising survival.

### 3.3. Role of MRI in Risk-Adapted Diagnostic Pathways

PCa screening began as a largely PSA-threshold–driven, one-size-fits-all pathway in which a raised PSA commonly led directly to systematic (non-targeted) biopsy. In ERSPC, repeated PSA screening reduced PCa mortality on long-term follow-up, but at the cost of substantial downstream testing and detection of cancers that would never have become clinically relevant. Interpretation of the Prostate, Lung, Colorectal and Ovarian Cancer Screening Trial (PLCO) has been complicated by opportunistic PSA testing in the control arm (“contamination”), so it compared organized screening versus a high background level of screening rather than screening versus no screening [12]. This early era therefore selected a broad population for biopsy, many men with benign disease, many with indolent GG1 cancers, and fewer with potentially lethal disease, producing the mortality benefit versus overdiagnosis/overtreatment trade-off (often estimated around 40–50% among screen-detected cases in ERSPC analyses) [13].

Risk stratification then evolved by inserting prediction models or biomarkers after PSA and before biopsy, shifting selection from a simple PSA cut-off toward an individualized probability of clinically significant PCa. Examples include ERSPC-derived risk calculators and biomarker-based models such as Stockholm3 and kallikrein panels [14]. These approaches preferentially triage men with higher predicted csPCa risk to biopsy while deferring biopsy in low-risk men despite modest PSA elevation, but when systematic biopsy remains the reference test, they remain vulnerable to undersampling and continued detection of low-grade disease, albeit at reduced rates versus PSA alone.

A major inflection point came with MRI as a triage test before biopsy, enabling targeted sampling. PROMIS established MRI as a useful triage test, and PRECISION showed that an MRI pathway with targeted biopsy increases detection of clinically significant PCa and reduces detection of clinically insignificant cancer versus standard systematic biopsy [9,15,16]. MRI-first strategies enrich for MRI-visible cancers and de-emphasize many men with PSA elevation but no MRI target, but can generate substantial numbers of MRIs in men who will not benefit and concentrate uncertainty in PI-RADS 3.

Pretreatment mpMRI risk strongly correlated with worse RP outcomes: higher PI-RADS independently predicted biochemical recurrence (BCR) in most studies, with reported HRs from about 1.75 to 6.19 depending on cutoff. After RP, men with PI-RADS 5 lesions had higher recurrence risk than those with lower-suspicion lesions; in one MRI-targeted biopsy cohort, PI-RADS 5 vs. 3 carried an HR of 2.86 for BCR [17]. In patients diagnosed with MRI-targeted biopsy (TBx) and treated by RP, adding PI-RADS score plus mpMRI features such as seminal vesicle invasion and lesion diameter improved 36-month BCR prediction versus clinical/TBx variables alone (73% vs. 66%).

Modern screening trials are increasingly using risk stratification to decide whether to perform an MRI. This involves a stepwise approach: starting with PSA-based entry and then enriching the risk for csPCa using biomarkers or models. Finally, MRI with targeted biopsy is reserved for men above a specific risk threshold. In ProScreen, a multi-step strategy (PSA → kallikrein panel risk score → MRI) detected additional high-grade cancers while minimizing additional low-grade detections [14]. Similarly, MRI-based extensions of Stockholm3 (STHLM3-MRI) were designed to reduce unnecessary biopsies and overdiagnosis by concentrating MRI and biopsy among men with a higher pretest probability of csPCa, based on integrated risk (clinical variables + biomarkers) [18].

**Figure 1:**
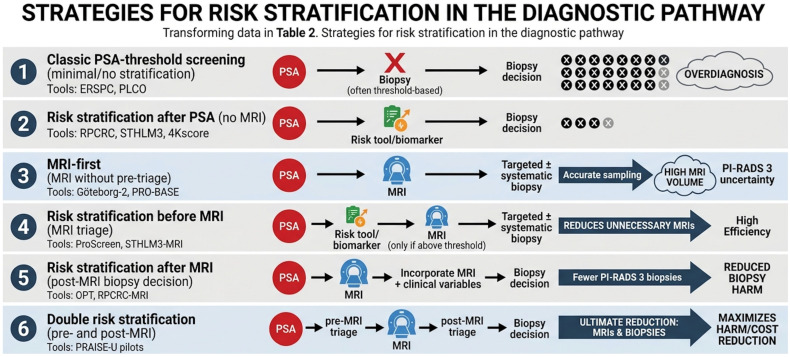
Strategies for risk stratification in the diagnostic pathway. PSA = Prostate-Specific Antigen; PCa = prostate cancer; csPCa = clinically significant prostate cancer; MRI = Magnetic Resonance Imaging;; RPCRC = Rotterdam Prostate Cancer Risk Calculator; STHLM3 = Stockholm3 model; ERSPC = European Randomized Study of Screening for Prostate Cancer; PLCO = Prostate, Lung, Colorectal, and Ovarian (Cancer Screening Trial); OPT = “optimal”/optimized post-test strategy (as used in some pathway descriptions; often referencing concepts like PSA density); PROBASE = Prostate Cancer Screening in Young Men (PROBASE trial); PRAISE-U = PRAISE-U pilots/trials (double risk-stratification pathway).

Risk stratification can also be applied after MRI, especially for MRI-negative and PI-RADS 3 cases, redefining “at risk” as those with meaningful residual csPCa probability. PSA density has been proposed as a post-MRI modifier, and evidence syntheses suggest that combining MRI findings with PSA density thresholds may reduce unnecessary biopsy while attempting to preserve sensitivity for csPCa; however, this approach should be interpreted cautiously given heterogeneity in the evidence base and limited validation across populations [19]. “Double risk stratification” formalizes layered pre-MRI enrichment and post-MRI refinement to reduce harms while maintaining early detection of lethal cancers, reflected in Europe’s stepwise policy approach (EU Council Recommendation, 9 December 2022) and initiatives such as PRAISE-U [20]. In Germany, PROBASE extends “risk stratification” to risk-adapted retesting intervals based on baseline PSA [21]. Figure 1 describes possible risk stratification strategies in the diagnostic pathway of PCa.

These shifts affect what is labelled “low-risk PCa” and how often it is treated: MRI-targeted and layered tools reduce random undersampling, making “low risk” more biologically consistent, while reducing detection of small indolent tumours can shrink the pool diagnosed with truly low-risk disease. In addition to imaging, molecular and liquid biomarkers are increasingly integrated into risk stratification pathways and are discussed in the following section.

### 3.4. Evolving Diagnostic Pathways: Biomarkers

In addition to these MRI pathways, biomarkers from blood, urine, and tissue (e.g., PCA3, Oncotype DX, Decipher) are being investigated to refine stratification and management, helping identify indolent disease versus risk of progression [16,22,23]. Table 2 summarizes possible applications and roles of these biomarkers in advancing the diagnostic and management pathway of Low-Risk PCa.

A meta-analysis of five studies showed that Decipher^®^ GC is an independent predictor of metastasis after radical prostatectomy (HR 1.30; 95% CI: 1.14–1.47; *p* < 0.001 per 0.1-unit increase), improving prognostic stratification across most clinicopathologic subgroups [24]. An RCT of 215 intermediate-risk PCa patients confirmed its prognostic value for disease progression (HR 1.12), biochemical failure (HR 1.22), distant metastasis (HR 1.28), and cancer-specific mortality (HR 1.45), though these were secondary endpoints [25].

Overall, while Decipher^®^ and Oncotype DX improve risk stratification, no definitive evidence confirms improved long-term oncologic outcomes. Selective use is reasonable when results may guide management, but routine use lacks prospective outcome data support. Their use may be considered selectively when results are likely to inform management, but routine use is not yet supported by prospective outcome data. The magnitude of their clinical benefit remains modest and heterogeneous across studies, and a definitive improvement in long-term oncologic outcomes has not yet been proven in prospective trials.

High-sensitivity liquid biopsy approaches, particularly circulating tumor DNA (ctDNA), are biologically appealing because they may capture occult molecular aggressiveness not reflected by conventional clinical variables. However, in localized low-volume disease, ctDNA remains constrained by low tumor shedding and limited analytical sensitivity; accordingly, current evidence does not support its routine use in low-risk active surveillance [26], and its role should presently be regarded as investigational, potentially within multimodal models or minimal residual disease-oriented settings rather than as a stand-alone decision tool. Beyond ctDNA, a broader range of diagnostic tools may help refine the timing of transition from surveillance to active treatment. These include blood-based markers, kallikrein-based panels, urine assays, tissue genomic classifiers, and imaging-derived risk stratification approaches, which may improve the identification of biologically significant disease among patients otherwise classified as low risk. In this context, the ProScreen randomized trial [14] supports the value of a multistep strategy integrating PSA, the kallikrein panel, and MRI, reinforcing the concept that future risk-adapted pathways will likely rely on combinations of complementary biomarkers rather than on any single parameter.

### 3.5. Active Surveillance and Risk-Adapted Follow-Up

AS is now the cornerstone of low-risk PCa management. It aligns treatment intensity with disease aggressiveness, achieving both cancer control and quality-of-life preservation. Multiple studies have established the long-term safety of AS, and with proper patient selection and monitoring protocols (including PSA kinetics, MRI, and periodic biopsies as needed), the risk of missing the “window of curability” is exceedingly low.

The ProtecT randomized trial in the UK [3], which followed mostly low-risk (and some intermediate-risk) patients for 15 years, found no difference in PCa-specific mortality between men randomized to active monitoring versus immediate surgery or radiation. At 15 years, PCa death rates remained very low (<3%) in all arms, confirming that surveillance does not compromise survival in low-risk disease.

A recent report from the Göteborg randomized screening trial provided up to 25-year outcomes for men managed with surveillance [27]. Among those with very-low-risk or low-risk PCa, the 20-year PCa-specific survival exceeded 90–99%. In the entire AS cohort (including some intermediate-risk patients), only 14 men (out of 488) died from PCa over a median 18-years follow-up. These data demonstrate that with vigilant monitoring, the likelihood of a low-risk patient dying of PCa is extremely low—typically 1–5% over two decades, often lower than competing health risks. Furthermore, about half of men on AS in such cohorts never require active treatment even after 15–20 years.

Long-term population-based data [28] support the use of risk-adapted management and deferred treatment strategies in men with low-risk disease. The 15-year PCa-specific mortality was only 5.5%, rising to 12% at 30 years. In contrast, the risk of death from other causes in this group reached 77% over the same 30-year period, indicating that most patients are far more likely to die with the disease than from it. Among men with a life expectancy greater than 15 years, 30-year PCa mortality remained low at 11%, reinforcing the safety of conservative approaches like AS.

Across contemporary AS cohorts and guideline-based pathways, follow-up intensity is typically front-loaded (to confirm eligibility) and then individualized over time. An example is the Johns Hopkins approach, which includes confirmatory biopsy within 6–24 months, PSA every 3–6 months, annual digital rectal examination (DRE), mpMRI every 2–3 years and repeat biopsies roughly every 1–5 years depending on interim findings [29].

In contrast, an older UCSF protocol emphasizes close PSA monitoring (every 3 months), interval prostate imaging with ultrasound (every 6–12 months), and biopsy every 1–2 years, reflecting how some long-running programs pre-dated today’s widespread mpMRI integration [30]. The Sunnybrook/University of Toronto schedule is often described as PSA every 3 months for 2 years, then every 6 months, with biopsy at 12 months and then every 3–4 years. Protocol-directed multicentre AS can be even more prespecified, as in Canary PASS (PSA every 3 months, DRE every 6 months, and repeat biopsies at 6–12, 24, 48, and 72 months) [31]. Guideline frameworks generally set upper bounds rather than mandating a single “best” cadence; for example, NCCN describes PSA no more often than every 6 months, DRE no more often than every 12 months, and repeat biopsy and mpMRI no more often than every 12 months (unless clinically indicated), with an early repeat biopsy within 6 months when the diagnostic sampling is limited or discordant. ASCO’s endorsement of Cancer Care Ontario similarly suggests confirmatory biopsy at 6–12 months and then serial biopsy every 2–5 years, alongside PSA every 3–6 months and at least annual DRE [32].

However, the implementation of these risk-adapted surveillance pathways is not uniform across healthcare systems. Access to high-quality mpMRI, expert radiological interpretation, and repeat biopsy strategies may be limited in resource-constrained settings, potentially leading to disparities in patient selection and monitoring intensity. In addition, the increasing incorporation of advanced tools such as genomic classifiers may further widen inequities due to cost and availability constraints. These factors may result in underuse of AS in some populations or, conversely, suboptimal monitoring where recommended protocols cannot be fully implemented.

De-escalation strategies are increasingly framed as risk-adapted “response-to-stability.” Long-term prospective data underscore why this matters: in Canary PASS, 49% of men remained free of progression or treatment at 10 years (with <2% metastatic disease and <1% PCa mortality), highlighting a sizable subgroup that may not need perpetual high-intensity testing [33]. A particularly actionable trigger for de-intensification is a negative follow-up biopsy, which in large multicenter data is associated with substantially lower risk of conversion to treatment (adjusted hazard ratio: 0.45; 95% CI: 0.42–0.49), and subsequent upgrading (adjusted HR for conversion ~0.45; adjusted OR 0.52; 95%CI: 0.45–0.62), supporting less invasive schedules in this subset [34]. In parallel, MRI can be used to rationalize intensity rather than replace tissue confirmation outright: the EAU notes that serial DRE may be omitted when MRI is stable, and that patients with low-risk disease, stable MRI, and low PSA density (<0.15) may be exempted from repeat biopsy when a repeat MRI is performed before the planned re-biopsy. Nevertheless, such MRI-driven de-escalation strategies presuppose consistent access to high-quality imaging, which may not be universally available, reinforcing the need to adapt protocols to local resources. Finally, de-escalation should also include “time horizon” considerations—NCCN explicitly states that repeat biopsies are not indicated when life expectancy is <10 years, supporting transition from AS to observation/watchful waiting in appropriately selected older or comorbid patients.

A comparative overview of active surveillance follow-up protocols across major institutions and guideline frameworks is provided in Table 3.

### 3.6. Beyond Surveillance: The Role of Focal Therapy

Focal therapy (FT) represents an evolving approach in PCa management, strategically positioned between AS and radical treatment [35,36]. Its definitive role remains to be established as higher-level evidence continues to emerge. FT is not intended to replace AS in genuine low-risk cases, which typically require no immediate intervention, nor should it substitute radical treatment in high-risk scenarios. Rather, FT serves as an intermediate solution for patients uncomfortable with surveillance, yet who do not necessitate immediate whole-gland therapy.

Patient selection is crucial, encompassing specific tumor paramenters (PSA, PSAD, ISUP at biopsy, tumor size, tumor location, prostate volume, and multifocality), patient characteristics (life expectancy, individual treatment goals, pelvic and prostate anatomy, acceptance of potential recurrence, and openness to retreatment or return to AS), and hospital organization (availability of advanced FT modalities, surgeon experience, quality of MRI imaging, and biopsy accuracy). However, selection criteria remain inconsistently defined across studies, contributing to variability in reported outcomes.

The ideal candidate for FT typically falls within the intermediate-risk category, balancing between sufficient disease progression risk to justify intervention and low likelihood of immediate metastatic progression or mortality. Such patients commonly have MRI-visible, biopsy-confirmed, clinically significant tumors spatially aligned for targeted ablation. However, this paradigm is challenged by the intrinsic multifocality of PCa and the limitations of imaging and biopsy in fully characterizing disease extent. Tumors located anteriorly or adequately distanced from critical structures (e.g., urethra, distal apex) represent optimal targets, while posterior lesions should preferably be small and safely situated. Nevertheless, the risk of undetected clinically significant disease outside the treatment field remains a key concern. Caution is warranted in younger men due to the uncertainty regarding FT’s long-term oncologic durability and its potential impact on subsequent salvage treatments, which remain insufficiently studied. Transition from AS to FT should be based on biopsy-confirmed progression, supported by imaging, rather than on any single parameter alone. The main triggers are grade upgrading from ISUP Grade Group 1 to csPCa, especially ISUP Grade Group ≥ 3, detection of cribriform or intraductal carcinoma, increasing tumor burden on repeat systematic biopsy, and MRI progression requiring confirmatory biopsy before treatment change. PSA kinetics alone should not trigger intervention. However, there is currently no standardized threshold for intervention, and decision-making remains partly subjective. When systematic and MRI-targeted biopsy grades are discordant, the associated risk is intermediate, not simply that of the highest grade alone: the rate of advanced pathological stage was 23% in concordant Grade Group 3 disease and 8.8% in concordant Grade Group 2 disease, compared with 18% for systematic Grade Group 3/targeted Grade Group 2 and 15% for the reverse combination [37]. This supports a more nuanced and individualized approach to treatment escalation. FT appears most appropriate in carefully selected patients with localized, MRI-visible, biopsy-confirmed csPCa but anatomically limited disease. However, available studies remain heterogeneous in patient selection, treatment platform, follow-up, and endpoint definition. FT for clinically localized PCa demonstrates meaningful short-term oncological control, with biopsy-proven csPCa recurrence-free survival (csPCa RFS) rates of 89%, 86%, and 81% at 6, 12, and 24 months, respectively, across 50 prospective studies including 4615 patients. However, comparative effectiveness data against AS remains sparse.

In the intermediate-risk subgroup—the most clinically relevant population—the 12-month csPCa RFS was 79%, confirming moderate cancer control even in more aggressive disease [38]. Five-year failure-free survival (radical and systemic treatment-free) reached 82%, suggesting that most patients can avoid whole-gland treatments at intermediate-term follow-up. However, these findings should be interpreted with caution, as most data derive from heterogeneous, single-arm studies with limited randomized comparisons, variable outcome definitions, and inconsistent follow-up protocols.

In-field and out-of-field csPCa recurrence rates were comparable (~9% and ~8%, respectively), highlighting the importance of careful patient selection given the multifocal nature of PCa [38]. Interestingly, no significant differences in oncological outcomes were observed across the seven energy sources investigated (HIFU, cryotherapy, IRE, FLA, PDT, focal brachytherapy, RFA). This apparent equivalence may reflect insufficient comparative power rather than true clinical similarity, and no modality has demonstrated clear superiority.

Treatment failure after FT should therefore be defined using composite criteria, including biopsy-confirmed persistent or recurrent csPCa, radiologic progression on follow-up MRI, and the need for salvage focal, whole-gland, or systemic treatment. At present, outcomes are strongly influenced by patient selection, operator experience, and institutional expertise, limiting generalizability. Though long-term data—including metastasis-free and overall survival—remain limited and are critically awaited [24,25], while AS remains the gold standard for low-risk (GG1) disease, focal therapy is increasingly positioned as a “middle ground” for favourable intermediate-risk (GG2) patients. It offers a higher chance of oncologic clearance than AS while significantly reducing the morbidity associated with radical prostatectomy or whole-gland radiation (Table 4).

**Figure 2:**
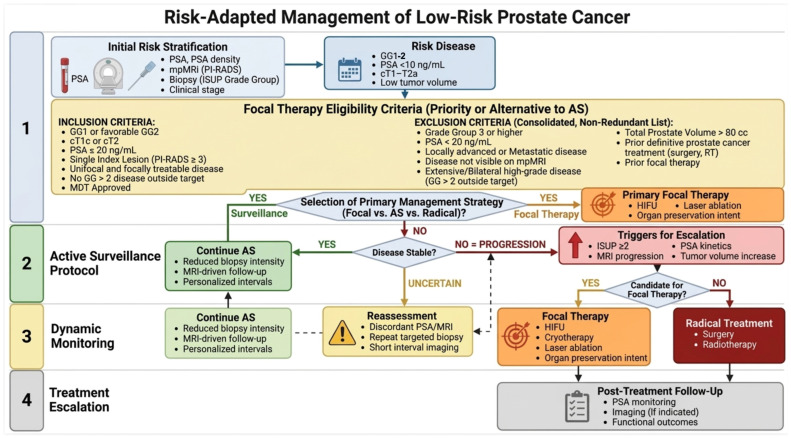
Risk-Adapted Management Algorithm for active surveillance and focal therapy.

To facilitate clinical implementation, a management algorithm integrating risk stratification, AS eligibility, monitoring, and escalation pathways is proposed (Figure 2). This framework reflects contemporary trends toward intensification and de-intensificaion while maintaining oncologic safety.

### 3.7. Current Limitations of the Diagnostic Pathway in Low-Risk Prostate Cancer

Despite significant strides in risk stratification and surveillance strategies, the diagnostic journey of low-risk PCa remains fraught with limitations that may affect optimal patient selection and long-term outcomes. These challenges encompass imaging and biopsy accuracy, risk of pathological upgrading, terminological controversies around GG1, and evolving genomic and histopathological markers.

MRI sensitivity remains variable, especially for small or low-grade lesions. Studies indicate that MRI may miss tumours <0.5 mL or those lacking aggressive histologic features, with sensitivity for index lesion detection ranging from 71% to 94% depending on technique and reader experience [39]. Additionally, interpretation inconsistencies across centers impact diagnostic yield. For example, biopsies informed by MRI from low-volume or less experienced centres show significantly reduced clinically significant PCa detection compared to high-volume centers [40]. Other imaging modalities like PET/CT are still considered experimental due to their limited value in intermediate and high-risk cases. While a positive correlation between SUV and pathological prostate features exists, PET/CT sensitivity remains a key limitation [41].

Biopsy strategies themselves present diagnostic gaps. A randomized trial comparing targeted biopsy (TB) alone versus TB plus systematic biopsy (SB) in MRI-positive, biopsy-naïve patients revealed that although TB alone suffices in detecting most csPCa, the addition of SB can improve overall detection by 7.3% [42]. These limitations underscore the persistence of sampling errors and false negatives even in the MRI era.

Despite initial classification as low risk, many patients experience histological upgrading upon repeat biopsy or post-prostatectomy analysis. In a contemporary cohort, 67.3% of low-risk patients demonstrated upgrading, with PSA levels and proportion of biopsy-positive cores emerging as independent predictors [43]. Similarly, long-term AS cohorts report a cumulative 10-year upgrading rate of 62%, with 19% showing major upgrading to GG ≥3 [44]. This risk raises concerns about the initial diagnostic accuracy and justifies the need for confirmation biopsies, especially for those not pre-stratified by MRI.

The introduction of MRI-guided targeted biopsies has contributed to a “stage shift” phenomenon. Lesions previously undetected with systematic biopsy are now identified, but this comes at the cost of reclassifying many patients into higher risk categories based on upgraded biopsy results. Consequently, the average risk profile of “low-risk” patients has evolved, complicating comparisons with historical cohorts and casting doubt on the current risk stratification thresholds. Indeed, MRI-targeted biopsies are more sensitive in detecting high-grade cancers but may also lead to a better prognosis within the same GG compared to systematic biopsies. As a result, the 2019 ISUP consensus conference recommended using an aggregated GG for MRI-targeted biopsy cores from the same lesion, rather than relying solely on the highest-grade core [45].

Whether GG1 represents true cancer remains controversial. Some propose relabelling it as a benign or “indolent neoplasm” to reduce anxiety and overtreatment [46]. However, a pathology society survey showed that 81.7% of experts oppose removing the “cancer” label, citing diagnostic reproducibility and potential confusion in management [47]. Critics argue that GG1 tumours share morphologic and molecular features with higher-grade cancers, and that removing the label could undermine proper monitoring.

Genomic classifiers such as Decipher and OncotypeDx have been integrated into modern risk stratification algorithms, offering potential additional prognostic value [10,22]. Yet, evidence from large prospective cohorts suggests that high-risk genomic profiles do not reliably predict upgrading on biopsy [44]. In multivariable models, traditional factors like PSA density and age retain stronger predictive value. The role of genomics remains adjunctive rather than definitive in the current diagnostic pathway.

The health-economic and implementation implications of genomic classifiers warrant discussion, as their cost-effectiveness and real-world utility remain debated. While these assays can add prognostic information beyond clinicopathologic factors, evidence that they consistently and meaningfully change routine management—and thereby improve patient-important outcomes—remains heterogeneous across settings and pathways. Cost-effectiveness is highly context-dependent and typically assumes that testing produces net de-escalation of unnecessary treatment or better-targeted escalation in selected subgroups; if instead it adds expense while only modestly shifting decisions (or increases downstream surveillance/utilization), economic value may be attenuated. Moreover, variable payer coverage, access, and workflow constraints shape uptake and may limit generalizability and equity [16].

Histologic findings such as Atypical Intraductal Proliferation (AIP) are emerging as critical yet underutilized predictors of unsampled high-grade disease [48]. AIP is frequently associated with adverse pathology and a higher likelihood of upgrading on repeat prostate biopsy. Despite this, AIP is not consistently reported in pathology or incorporated into surveillance protocols. Its routine documentation could enhance risk stratification, guiding more tailored monitoring strategies.

## 4. Conclusions

The management of low-risk PCa is a success story of risk adaptation in oncology. AS has established itself as the standard of care for low-risk ISUP I PCa, supported by long-term cohort data and randomized trial evidence demonstrating oncologic safety without compromising functional outcomes. Refined risk stratification tools—including mpMRI, biomarkers, and genomic classifiers—have further reduced overtreatment, though their optimal integration into clinical pathways remains to be standardized. FT represents a promising minimally invasive alternative, yet critical evidence gaps persist: long-term cancer control durability, out-of-field progression risk, implications for salvage treatments, and absence of comparative effectiveness data against AS or radical therapy. To bridge the gap between evidence and clinical practice, a risk-adapted management approach should integrate initial risk stratification, AS eligibility and follow-up protocols, de-escalation pathways, and escalation triggers, providing a structured decision-making framework applicable across different healthcare settings.

From a clinical standpoint, key priorities include: (i) adherence to guideline-recommended AS protocols for low-risk disease; (ii) structured use of mpMRI for dynamic monitoring; and (iii) restriction of FT to clinical trials or prospective registries until robust long-term data are available.

Future research should focus on head-to-head prospective comparisons of AS and FT, standardization of oncologic and patient-reported outcome measures, and validation of integrated risk models combining clinical, imaging, and molecular data. Real-world registry-based evidence will be essential to define the evolving role of minimally invasive approaches and to ensure equitable, resource-adapted implementation across diverse healthcare systems.

## Figures and Tables

**Table 1:** Search strategy summary.

Items	Specifications
Databases and other sources searched	PubMed/MEDLINE, Embase, Scopus, Cochrane Library + manual reference screening
Search terms used	low-risk prostate cancer, active surveillance, MRI, biomarkers, focal therapy, overtreatment
Timeframe	2014–2025
Inclusion criteria	Studies on localized low-risk PCa (GG1, PSA ≤ 10, cT1–T2a), including trials, cohorts, registries, and guidelines
Selection process	Two authors independently performed data collection; discrepancies were resolved with a third author; final synthesis validated by expert panel

**Table 2:** Comparative tools for translational evaluation of “low-risk” prostate cancer.

Tool Category	Specific Tests	Pathway Integration	Clinical Utility	Strategic Advantage	Critical Limitations
Liquid Biopsy (Serum/Urine)	PHI, 4Kscore, S3M, SelectMDx, ExoDx (EPI)	Pre-Biopsy/Pre-MRI Triage	Refines the “gray zone” PSA signal; provides a probability of clinically significant PCa (csPCa, GG ≥ 2).	Reduces unnecessary MRI and biopsy burden; non-invasive.	Significant cost; potential for geographic/population calibration drift.
Morphometric and Advanced Imaging	PSAD, mpMRI (PI-RADS 2.1), Micro-ultrasound (MicroUS)	Pre-Biopsy/Diagnostic Localization	PSAD: PSA/V prostate. Imaging: Localizes lesions to transition from systematic to targeted sampling.	Improves detection of csPCa while minimizing GG1 (overdiagnosis).	High inter-reader variability; MRI access and infrastructure constraints.
Molecular Imaging	PSMA PET/CT (intraprostatic)	Adjunct to MRI/Equivocal Triage	Provides biologic signal (PSMA expression) where anatomy (MRI) is uncertain.	High specificity for high-grade disease; helps resolve MRI-negative suspicion.	High cost; radiation exposure; evolving role in primary diagnosis.
Epigenetic Tissue Analysis	ConfirmMDx	Post-Negative Biopsy Triage	Identifies DNA methylation “field effects” to detect cancer missed by the needle.	Addresses sampling error/undersampling; reduces unnecessary repeat biopsies.	Requires existing tissue; not a primary grading or prognostic tool.
Genomic Classifiers (Tissue-based)	GPS, CCP, Decipher	Post-Diagnosis/Treatment Selection	Analyzes RNA expression to distinguish “true low-risk” from “aggressive” biology.	Supports AS vs. intervention; predicts metastatic risk.	Not recommended for routine use; selective use based on clinical uncertainty.

PSA, prostate-specific antigen; PSAD, PSA density; PHI, Prostate Health Index; S3M, Stockholm3 model; EPI, Exosomal RNA-based Prostate Intelliscore; GPS, Genomic Prostate Score; CCP, Cell Cycle Progression; mpMRI, multiparametric magnetic resonance imaging; PI-RADS v2.1, Prostate Imaging Reporting and Data System version 2.1; MicroUS, micro-ultrasound; PSMA, prostate-specific membrane antigen; PET/CT, positron emission tomography/computed tomography; PCa, prostate cancer; csPCa, clinically significant prostate cancer; GG, Grade Group; AS, active surveillance.

**Table 3:** Comparison of Active Surveillance Follow-up Protocols across major institutions and guidelines.

Institution/Guideline	PSA	DRE	MRI	Biopsy	Trigger for Intervention	Notes
Johns Hopkins	Every 3–6 months	Yearly	Every 2–3 years	1–5 years (risk-adapted)	Upgrade to ≥GG2, PSA kinetics, MRI progression	Early confirmatory biopsy; highly structured protocol
UCSF	Every 3 months	Not standardized	Variable	Every 1–2 years	Pathologic upgrade, PSA rise	Older protocol, pre-MRI era
Sunnybrook (Toronto)	q3 months (2 yrs), then q6 months	Variable	Selective	12 months, then q3–4 years	Upgrade, volume progression, PSA kinetics	Widely adopted risk-adapted model
Canary PASS	Every 3 months	Every 6 months	Not mandatory	6–12, 24, 48, 72 months	Protocol-defined progression criteria	Prospective multicenter protocol
NCCN	≤every 6 months	≤yearly	As indicated	≤yearly (early confirmatory)	Upgrade, clinical progression	Upper limits rather than fixed schedule
EAU	Risk-adapted	Risk-adapted	Increasing role	Risk-adapted	Upgrade, MRI progression, PSA density	MRI-driven de-escalation strategies

PSA = Prostate-Specific Antigen; DRE = digital rectal examination; MRI = Magnetic Resonance Imaging; GG = Grade Group; AS = active surveillance; NCCN = National Comprehensive Cancer Network; EAU = European Association of Urology; UCSF = University of California, San Francisco; PASS = Prostate Active Surveillance Study.

**Table 4:** Comparison of focal therapies, their selected patient indications and therapeutic outcomes.

Modality	Typical Patient Selection	Mechanism	Reported Oncologic Outcomes *	Functional Outcomes	Key Limitations
HIFU	Intermediate-risk (GG2–3), MRI-visible, localized; apical lesions preferred.	Thermal ablation via high-intensity focused ultrasound.	csPCa-free survival ~75–85% at 2 yrs; FFS ~70% at 5 yrs.	High continence (~95%); Moderate ED risk (20–30%).	Difficulty treating large glands or calcifications; operator-dependent.
Cryotherapy	Intermediate-risk; localized; better for posterior/peripheral lesions.	Freeze–thaw cycles (Argon/Helium) causing ice ball necrosis.	Similar to HIFU; ~75% 5-yr biochemical recurrence-free survival.	Good continence; Higher ED risk than HIFU due to ice ball spread.	Risk of rectal fistula (though rare with warming probes); heterogeneous data.
IRE	Lesions also near critical structures (e.g., urethra, neurovascular bundle).	Non-thermal permanent cell membrane pore formation (electroporation).	Early series: 80% control at 2 yrs; limited long-term data.	Superior preservation of nerves and collagenous structures.	Requires general anesthesia and muscle paralysis; high cost.
FLA	Small, MRI-visible, low–intermediate risk (GG1–2) lesions.	Laser-induced thermotherapy causing coagulative necrosis.	~70–80% short-term (1 yr) biopsy-free rate.	Excellent; minimal impact on urinary or sexual function.	Small treatment zones; high rate of “in-field” recurrence in some series.
VTP (PDT)	Low-risk (GG1) or favorable intermediate-risk.	Vascular-targeted light-activated cytotoxic reaction (e.g., Tookad).	RCT shows ~50% reduction in progression to radical RX vs. AS.	Very high functional preservation; mild transient urinary symptoms.	Requires specialized laser fibers and light-shielding post-op.
Focal Brachy	Intermediate-risk (GG2); localized and well-defined.	Localized radiation (LDR seeds or HDR temporary needles).	High local control; comparable to whole-gland brachytherapy.	Moderate urinary irritative symptoms; gradual decline in ED over time.	Late radiation toxicity; “focal” boundaries are less distinct than ablation.
RFA	Small, localized lesions; primarily in research settings.	Thermal ablation via high-frequency alternating current.	Sparse; early data suggests ~70% short-term success.	Likely favorable; similar to FLA or HIFU.	Very limited data; largely superseded by HIFU and Cryo in the US/EU.

* csPCa: Clinically significant Prostate Cancer (typically defined as Grade Group 2); FFS: Failure-Free Survival (avoidance of salvage radical treatment or systemic therapy); ED: Erectile Dysfunction; AS (Active Surveillance); IRE—Irreversible Electroporation; FLA—Focal Laser Ablation; VTP (PDT)—Vascular-Targeted Photodynamic Therapy (Photodynamic Therapy); Focal Brachy—Focal Brachytherapy; RFA—Radiofrequency Ablation.

## Data Availability

No new data were created or analyzed in this study. Data sharing is not applicable to this article.

## Supplementary Materials

The following supporting information can be downloaded at: https://www.mdpi.com/article/10.3390/diagnostics16091310/s1.
