# Supplementary material for: Beyond One-Size-Fits-All Active Surveillance for Low-Risk Prostate Cancer: Risk-Adapted Follow-Up, De-Escalation Pathways, and Focal Therapy as Tailored Strategy
Source: Diagnostics (Basel). 2026 Apr 27;16(9):1310. doi: 10.3390/diagnostics16091310 (PMC13162911; doi:10.3390/diagnostics16091310)
Supplement: Supplementary file 1 [file diagnostics-16-01310-s001.zip › diagnostics-4192876-supplementary.pdf]

10.3390/diagnostics16091310Simone Albisinni
Urology Unit, Department of Surgical Sciences, University of Rome Tor Vergata, Rome, Italy
Daniele Amparore
Department of Oncology, University of Turin, Orbassano, Turin, Italy
Division of Urology, Department of Surgery, Candiolo Cancer Institute, FPO-IRCCS, Candiolo (TO), Italy
Riccardo Giuseppe Bertolo
Urology Unit, AOUI Verona, University of Verona, Verona, Italy
Lorenzo Bianchi
Division of Urology, IRCCS Azienda Ospedaliero-Universitaria di Bologna, Bologna, Italy
Department of Medical and Surgical Sciences (DIMEC), University of Bologna, Bologna, Italy
Riccardo Campi
Department of Experimental and Clinical Medicine, University of Florence, Florence, Italy
Unit of Urology and Renal Transplantation, Oncology Department, Careggi University Hospital, Florence, Italy
Department of Urology, Comprehensive Cancer Center, Medical University of Vienna, Vienna, Austria
Roberto Contieri
Department of Urology, Istituto Nazionale Tumori IRCCS Fondazione G. Pascale, Naples, Italy
Elisa De Lorenzis
Department of Clinical Sciences and Community Health, University of Milan, Italy
Department of Urology, Foundation IRCCS Ca' Granda - Ospedale Maggiore Policlinico, Milan, Italy
Paolo Dell'Oglio
Department of Urology, ASST Grande Ospedale Metropolitano Niguarda, Milan, Italy
Interventional Molecular Imaging Laboratory, Department of Radiology, Leiden University Medical Center, Leiden, The Netherlands
Ettore Di Trapani
Department of Urology, IRCCS European Institute of Oncology (IEO), Milan, Italy
Francesco Esperto
Research Unit of Urology, Department of Medicine and Surgery, Università Campus Bio-Medico di Roma, Rome, Italy
Department of Urology, Fondazione Policlinico Universitario Campus Bio-Medico, Rome, Italy
Ugo Giovanni Falagario
Department of Urology and Kidney Transplantation, University of Foggia, Foggia, Italy
Department of Molecular Medicine and Surgery, Karolinska Institutet, Stockholm, Sweden
Department of Medical Epidemiology and Biostatistics, Karolinska Institutet, Stockholm, Sweden
Andrea Mari
Unit of Oncologic Minimally-Invasive Urology and Andrology, Department of Experimental and Clinical Medicine, Careggi Hospital, University of Florence, Florence, Italy
Giancarlo Marra
Division of Urology, Department of Surgical Sciences, University of Turin and Città della Salute e della Scienza, Turin, Italy
Michele Marchioni
UniCamillus-Saint Camillus International University of Health Sciences, Rome, Italy
Department of Urology, Leonardo Foundation, Abano Terme, Italy
Veronica Mollica
Medical Oncology, IRCCS Azienda Ospedaliero-Universitaria di Bologna, Bologna, Italy
Marco Moschini
Department of Urology, IRCCS San Raffaele Scientific Institute, Milan, Italy
Vita-Salute San Raffaele University, Milan, Italy

Savio Domenico Pandolfo
Department of Life, Health and Environmental Sciences, University of L'Aquila, Italy
Department of Neurosciences, Reproductive Sciences and Odontostomatology, University of Naples Federico II, Naples, Italy
Pietro Piazza
Division of Urology, IRCCS Azienda Ospedaliero-Universitaria di Bologna, Bologna, Italy
Department of Medical and Surgical Sciences (DIMEC), University of Bologna, Bologna, Italy
Francesco Prata
Department of Urology, Fondazione Policlinico Universitario Campus Bio-Medico, Rome, Italy
Research Unit of Urology, Department of Medicine and Surgery, Università Campus Bio-Medico di Roma, Rome, Italy
Stefano Puliatti
Division of Urology, University of Modena and Reggio Emilia, Italy
Francesco Soria
Division of Urology, Department of Surgical Sciences, University of Turin and Città della Salute e della Scienza, Turin, Italy
Michele Talso
Department of Urology, ASST Fatebenefratelli Sacco, Milan, Italy
Filippo Turri
Department of Urology, Fondazione Policlinico Universitario Agostino Gemelli IRCCS, Università Cattolica del Sacro Cuore, Rome, Italy
Fabio Zattoni
Urologic Unit, Department of Surgery, Oncology and Gastroenterology, University of Padova, Padua, Italy
